# Purine metabolism in plant pathogenic fungi

**DOI:** 10.3389/fmicb.2024.1352354

**Published:** 2024-02-07

**Authors:** Manli Sun, Pengbo Dai, Zhiyan Cao, Jingao Dong

**Affiliations:** State Key Laboratory of North China Crop Improvement and Regulation/Key Laboratory of Hebei Province for Plant Physiology and Molecular Pathology/College of Plant Protection, Hebei Agricultural University, Baoding, Hebei, China

**Keywords:** purine *de novo* pathway, purine salvage pathway, purine catabolic pathway, plant pathogenic fungi, pathogenesis, purine transporters

## Abstract

In eukaryotic cells, purine metabolism is the way to the production of deoxyribonucleic acid (DNA) and ribonucleic acid (RNA) and plays key roles in various biological processes. Purine metabolism mainly consists of *de novo*, salvage, and catabolic pathways, and some components of these pathways have been characterized in some plant pathogenic fungi, such as the rice blast fungus *Magnaporthe oryzae* and wheat head blight fungus *Fusarium graminearum*. The enzymatic steps of the *de novo* pathway are well-conserved in plant pathogenic fungi and play crucial roles in fungal growth and development. Blocking this pathway inhibits the formation of penetration structures and invasive growth, making it essential for plant infection by pathogenic fungi. The salvage pathway is likely indispensable but requires exogenous purines, implying that purine transporters are functional in these fungi. The catabolic pathway balances purine nucleotides and may have a conserved stage-specific role in pathogenic fungi. The significant difference of the catabolic pathway *in planta* and *in vitro* lead us to further explore and identify the key genes specifically regulating pathogenicity in purine metabolic pathway. In this review, we summarized recent advances in the studies of purine metabolism, focusing on the regulation of pathogenesis and growth in plant pathogenic fungi.

## 1 Introduction

Purines, one of the most abundant metabolites in eukaryotic cells, participate in a series of basic cellular processes. In addition to serving as building blocks of DNA and RNA, purine nucleotides play essential roles in cellular energy homeostasis and signal transduction. Purines can also be converted into other molecules to serve as cofactors, such as coenzyme A and NAD, to well regulate biochemical reactions.

Purine metabolism is achieved by three pathways: *de novo* pathway, salvage pathway, and catabolic pathway, all of which involve a series of well-regulated sequential biochemical reactions with metabolite conversion (Zrenner et al., 2006). New purine bases are obtained mainly by *de novo* and salvage pathways. Purine *de novo* pathway is well-conserved in the eukaryotic cells and consists of three branches of inosine monophosphate (IMP), adenosine monophosphate (AMP), and guanosine monophosphate (GMP) *de novo* synthesis. Purine salvage pathway recycles preformed nucleosides and nucleobases. To maintain the metabolic balance, purine metabolites need to be degraded by catabolic pathway, through which the nucleotides are dephosphorylated into nucleosides, and subsequently hydrolyzed into ribose and bases (Tozzi et al., 2006).

In eukaryotic organisms, purine metabolic pathways play crucial roles in promotion of growth, development and pathogenesis. In plant pathogenic fungi, various genes related to the *de novo* and salvage purine synthesis as well as purine catabolism have been functionally characterized. This review aims to summarize key observations from these studies and discuss the importance of purine metabolism in growth, development, and pathogenesis in necrotrophic, hemibiotrophic, and biotrophic pathogens.

## 2 Purine metabolism in *Saccharomyces cerevisiae*

Among eukaryotic organisms, purine metabolism is best characterized in the budding yeast *Saccharomyces cerevisiae* (Table 1), a model in which metabolic pathways are comprehensive described. In yeast, purine metabolism is orchestrated by purine *de novo*, salvage, and catabolic pathways, and each of them has different roles in regulating growth and development (Ljungdahl and Daignan-Fornier, 2012).

**Table 1:** Key components of purine metabolic pathways in *F. graminearum*, and their orthologs in *M. oryzae* and *S. cerevisiae*.

	*F. graminearum*	*M. oryzae*	*S. cerevisiae*	Functions
Purine *de novo* pathway	FgAde4 (FGSG_05278^a^)	MoAde4	Ade4	Phosphoribosylpyrophosphate amidotransferase (PRPPAT)
	FgAde5,7 (FGSG_02506^a^)	MGG_11343^a^	Ade5,7	Aminoimidazole ribonucleotide synthetase and glycinamide ribotide synthetase
	FgAde8 (FGSG_08429^a^)	MoAde8	Ade8	Phosphoribosyl-glycinamide transformylase
	FgAde6 (FGSG_09440^a^)	MoAde6	Ade6	Formylglycinamidine-ribonucleotide (FGAM)-synthetase
	FgAde2 (FGSG_10669^a^)	MGG_01256^a^	Ade2	Phosphoribosylaminoimidazole carboxylase
	FgAde1 (FGSG_09453^a^)	MoAde1	Ade1	N-succinyl-5-aminoimidazole-4-carboxamide ribotide synthetase
	FgAde13 (FGSG_09185^a^)	MGG_03645^a^	Ade13	Adenylosuccinate lyase
	Acd16	MGG_04435^a^	Ade16, Ade17	AICAR transformylase and IMP cyclohydrolase
	FgAde12	MGG_17000^a^	Ade12	Adenylosuccinate synthase
	FgImd1	MoImd4	Imd1-4	*IMD2-4* genes encode AICAR transformylase and IMP cyclohydrolase, *IMD1* is a pseudogene.
	FgGua1 (FGSG_10358^a^)	MGG_00919^a^	Gua1	GMP synthase
	FgAdk1 (FGSG_10737^a^)	MGG_01058^a^	Adk1	Adenylate kinase
	FgAdk2 (FGSG_09162^a^)	MGG_03683^a^	Adk2	Adenylate kinase
	FgAdo1 (FGSG_06932^a^)	MGG_06270^a^	Ado1	Adenosine kinase
	FgGuk1 (FGSG_05956^a^)	MoGuk1	Guk1	Guanylate kinase; converts GMP to GDP
	FgNdk1 (FGSG_05972^a^)	MoNdk1	Ynk1/Ndk1	Nucleoside diphosphate kinase
Purine salvage pathway	FgApt1	MGG_17399^a^	Apt1, Apt2	Adenine phosphoribosyltransferase
	FgHpt1	MGG_10052^a^	Xpt1	Xanthine-guanine phosphoribosyl transferase
			Hpt1	Dimeric hypoxanthine-guanine phosphoribosyltransferase;
	Acd3	–	Aah1	Adenine deaminase (adenine aminohydrolase)
	Acd14	MGG_04119^a^	Gud1	Guanine deaminase
Purine catabolic pathway	Acd1	MGG_05630^a^	Amd1	AMP deaminase
	–	–	Pnp1	Purine nucleoside phosphorylase
	FgIsn1 (FGSG_11629^a^)	MGG_02955^a^	Isn1	Inosine 5′-monophosphate (IMP)-specific 5′-nucleotidase
	FgPhm8 (FGSG_06179^a^)	MGG_01783^a^	Phm8, Sdt1	Lysophosphatidic acid (LPA) phosphatase, nucleotidase

–, No homolog.^a^Predicted gene number (Homolog is present but not characterized).

Purine *de novo* pathway consists of three branches including IMP, AMP and GMP *de novo* synthesis. In yeast, purine *de novo* pathway originates from 5-phosphoribosyl-1-pyrophosphate (PRPP) to produce the first purine nucleotide IMP by 10 enzymatic steps. IMP has an intermediate role as the precursor used for both AMP and GMP synthesis (Vavassori et al., 2005; Rolfes, 2006). Deletion of *ADE16* and *ADE17*, two paralogous genes encoding the enzymes that catalyze the last two steps of IMP *de novo* synthesis from PRPP, resulted in adenine and histidine auxotrophy, and *ADE16* may plays a role in respiration and sporulation in *S. cerevisiae* (Tibbetts and Appling, 2000). In addition, accumulation of 5-phosphoribosyl-5-amino-4-imidazole carboxamide ribonucleotide (AICAR) interferes methionine biosynthesis and other cellular processes when both *ADE16* and *ADE17* are deleted in the budding yeast (Rébora et al., 2005).

IMP formed by purine *de novo* synthesis is then converted to AMP and GMP, respectively (Rolfes, 2006). In this process, the rate-limiting reaction from IMP to XMP is catalyzed by IMP dehydrogenase (Imd) (Hyle et al., 2003). There are four *IMD* (*IMD1-4*) genes in *S. cerevisiae*, among them, *IMD1* is a pseudogene, and Imd2 is intrinsically resistant to drug which could be significantly induced upon mycophenolic acid (MPA) treatment. Both *IMD3* and *IMD4* could not confer MPA resistance to cells lacking *IMD2*. Moreover, deletion of either of *ScIMD* family member confers yeast cells guanine auxotrophy (McPhillips et al., 2004).

*S. cerevisiae* could take up extracellular purine bases and re-utilize nucleosides and nucleotides through the salvage pathway (Kokina et al., 2019). In this process, xanthine-guanine phosphoribosyl transferase (Xpt1) is required for xanthine utilization and optimally utilization of guanine (Guetsova et al., 1999), hypoxanthine-guanine phosphoribosyltransferase (Hpt1) is used to convert hypoxanthine and guanine to IMP and GMP, respectively (Lecoq et al., 2000), and adenine phosphoribosyltransferase (Apt1) is recruited to convert adenine into AMP (Alfonzo et al., 1995).

In yeast, purine catabolic reactions ensure the balanced homeostasis of preformed nucleosides and nucleotides. High-energy demand leads to a high ATP availability by irreversibly degrading AMP using AMPD (AMP deaminase) (Sims et al., 1999). Conversion of AMP to IMP is the sole pathway of AMP catabolism in eukaryotic cells (Meyer et al., 1989; Saint-Marc et al., 2009). Yeast AMP deaminase Amd1 is responsible for AMP catabolism, and the deletion of Amd1 shows sporulation defect and exhibits hyper-accumulation of ATP and depletion of guanosine nucleotides (Walther et al., 2014).

## 3 Importance of IMP *de novo* synthesis in plant pathogenic fungi

**Figure 1:**
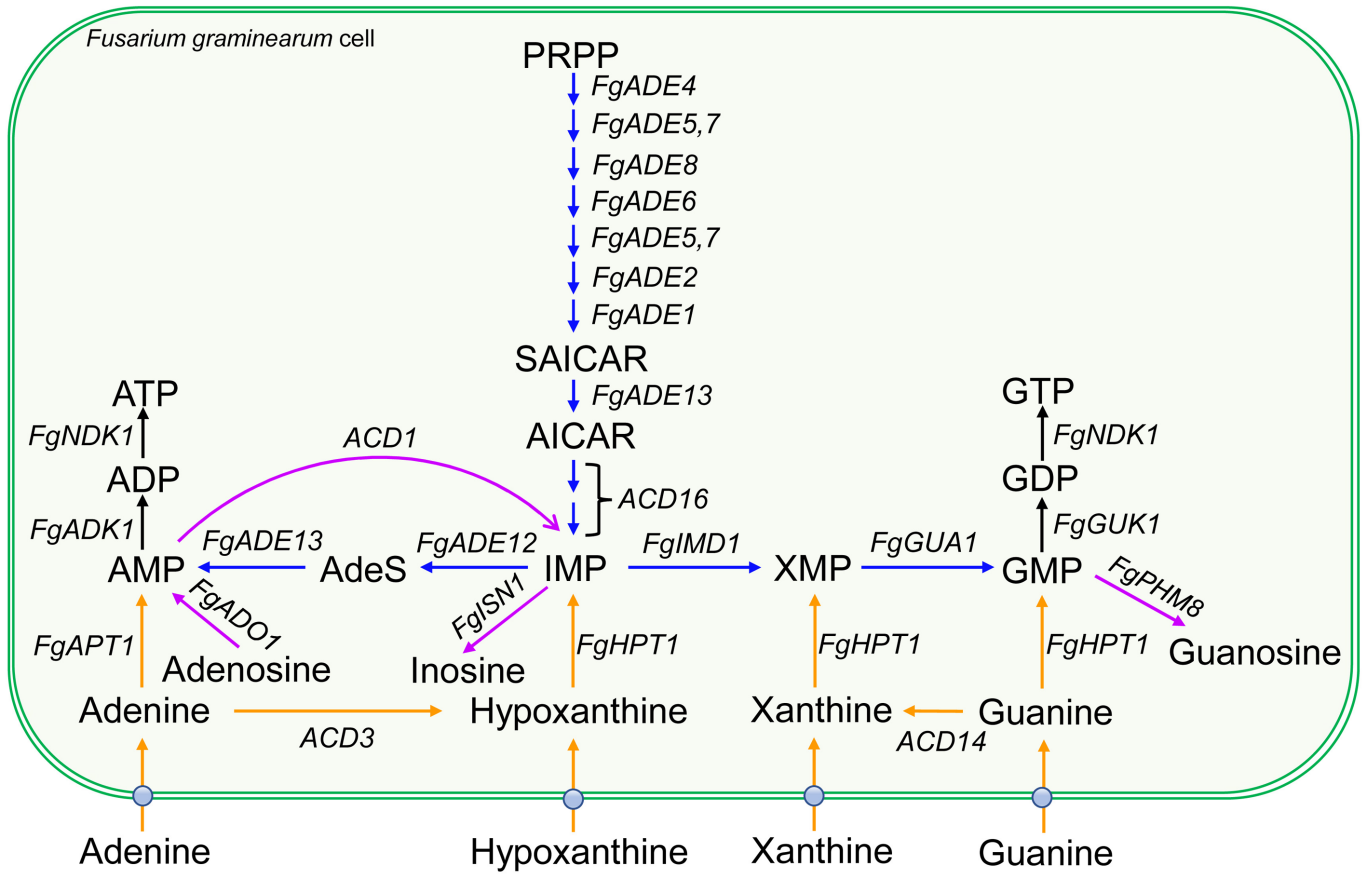
Purine *de novo*, salvage and catabolic pathways in *Fusarium graminearum*. Blue arrows represent the steps of purine *de novo* biosynthesis pathway. Orange arrows represent the steps of purine salvage biosynthesis pathway. The purple arrow indicates the degradation steps for purine catabolism. The ball on fungal plasma membrane indicates purine transporters. Gene names are italicized. AICAR, 5-phosphoribosyl-5-amino-4-imidazole carboxamide ribonucleotide; AdeS, adenylosuccinate; PRPP, 5-phosphoribosyl-1-pyrophosphate; SAICAR, 5-phosphoribosyl-4-(N-succinocarboxamide)-5-amino-imidazole ribonucleotide. The gene ID for each gene is listed in Table 1.

Dysfunction of *de novo* pathway could result in the defects of vegetative growth and plant infection (Fernandez et al., 2013; Sun et al., 2021; Aron et al., 2022). Among plant pathogenic fungi, the purine *de novo* pathways are mostly characterized in two hemibiotrophic pathogens, rice blast fungus *Magnaporthe oryzae* and wheat head blight fungus *Fusarium graminearum* (Figure 1), both of which are ranked in the top 10 fungal pathogens in molecular plant pathology (Dean et al., 2012). Genes in the IMP *de novo* pathway play important roles in various developmental processes (Table 1), and are related to the pathogenicity in many cases.

In *Coniothyrium minitans*, the first enzyme for purine *de novo* biosynthesis phosphoribosylpyrophosphate amidotransferase (PRPPAT) was firstly proved to be required for conidiation (Qin et al., 2011). In *M. oryzae*, *MoADE4* encodes the corresponding enzyme catalyzing the first step for the conversion of PRPP into 5-phosphoribosyl-1-amine (PRA). The deletion of *MoADE4* results in adenine, adenosine, and hypoxanthine auxotrophy on minimal medium. Interestingly, the conidia of *Moade4* mutant could form appressoria but fails to penetrate the cuticle of rice and ultimately results in completely nonpathogenic on rice and barley leaves. Moreover, exogenous adenine could partially rescue conidiation, infectious growth, and pathogenicity defects (Aron et al., 2022). The hosphoribosylglycinamide formyltransferase Ade8 is functionally downstream of Ade4, catalyzing the third step of purine *de novo* pathway, and the deletion of *ADE8* could cause defects in vegetative growth, conidiation, and pathogenicity (Liu et al., 2022). Because appressoria fail to penetrate host cells, *Moade8* mutant shows obvious defects in virulence on rice and barley, and exogenous adenine could not rescue the appressoria defect (Liu et al., 2022). All these results indicate the key important roles of *ADE8* in *M. oryzae*. However, *ADE8* is not always indispensable, for example, in *Candida albicans*, a principal human fungal pathogen, *ADE8* is conditionally essential and can be bypassed when exogenous adenine exists (Jiang et al., 2010). In addition, mutants of the other two genes (*MoADE5,7* and *MoADE6*) involved in IMP *de novo* pathway show similar defects to *Moade8* (Liu et al., 2022). Not only that, Ade6 catalyze the downstream reaction of Ade8 and was proved to be required for exogenous adenine in biotrophic pathogen *Ustilago maydis*, an ubiquitous smut fungus infecting maize (Heidenreich et al., 2008). As is reported, MoAde1, an ortholog of yeast Ade1 which is responsible for the seventh step for converting 4-carboxy-5-aminoimidazole ribonucleotide (CAIR) to 5-phosphoribosyl-4-(N-succinocarboxamide)-5-amino-imidazole ribonucleotide (SAICAR), is required for vegetative growth and pathogenicity. Different from the *Moade4* mutant, the *Moade1* mutant could form functional appressoria to penetrate host cells, but notably reduced for hyphae growth *in planta* (Fernandez et al., 2013). These studies in *M. oryzae* laid the foundation for research of IMP *de novo* pathway in plant pathogenic fungi.

The defects of *ade16 ade17* double mutant in *S. cerevisiae* are consistent with disruption of the AICAR transformylase/IMP cyclohydrolase (ATIC) encoded by the gene *ADE16* in *Cryptococcus neoformans* which is a fungal pathogen causing meningoencephalitis in the immunocompromised (Tibbetts and Appling, 2000; Wizrah et al., 2022). In *C. neoformans*, *ADE16* deletion resulted in adenine and histidine auxotrophs and lead to the block of the establishment of infection to a murine model (Wizrah et al., 2022). Similar to *C. neoformans*, *F. graminearum* also has only one orthologous gene (*ACD16*) of yeast *ADE16* and *ADE17*. The deletion mutant of *ACD16* has severe defects in vegetative growth, conidiation, sexual reproduction, and plant infection. When supplied with exogenous adenine and histidine, the vegetative growth could be rescued, but the perithecia formation and plant infection could not. Because *acd16* mutant fails to form an infection cushion to penetrate the host cell, it is nonpathogenic on wheat spikelets and corn silks (Sun et al., 2021). The two-step reaction from AICAR to IMP catalyzed by Ade16 and Ade17 in the budding yeast corresponds to the sole reaction catalyzed by Acd16 in *F. graminearum* and the enzyme encoded by MGG_04435 in *M. oryzae* (Table 1), respectively. Apart from this reaction, the purine *de novo* pathway is well conserved in these two plant pathogenic fungi. These findings suggest that IMP *de novo* pathway has a critical role in pathogenicity and growth of plant pathogenic fungi.

Collectively, these discoveries have shown that IMP *de novo* pathway is functionally well-conserved in plant pathogenic fungi, because all the genes involved in this pathway are crucial for vegetative growth and virulence with the common characteristic that the infection structures are almost defective in penetration and lead to nearly nonpathogenic to the host, even though the infectious processes vary among different fungi. The results in *M. oryzae* and *F. graminearum* shed light on that *de novo* synthesis is functionally conserved in growth, development and pathogenesis in plant pathogenic fungi.

## 4 Roles of AMP and GMP *de novo* synthesis in fungal pathogens

Two key genes functioning in AMP and ADP *de novo* pathways, *ADE12* and *ADK1*, have been shown to be tightly linked to the pathogenicity in phytopathogens. In *M. oryzae, MoADE12* encoding an adenylosuccinate synthase is important for the conidiation, sexual reproduction, and pathogenicity. The *Moade12* mutant does not affect appressoria function but has defects in infectious hyphae growth, indicating that *MoADE12* is essential for invasive growth in *M. oryzae* (Zhang et al., 2022). In *F. graminearum*, the *Fgade12* deletion mutant has pleiotropic defects in vegetative growth, conidiation, sexual reproduction, and pathogenicity (Sun et al., 2021). The functional characterization of *ADK1* in *M. oryzae* is still lacking, however, Su et al. (2020) showed that the deletion mutant of *ADK1* in the cotton Verticillium wilt fungus *Verticillium dahlia*, a hemibiotrophic pathogen, has reduced virulence on the host plants. More interestingly, host-induced gene silencing (HIGS) of the gene coding adenylate kinase improved plant resistance to *V. dahlia* (Su et al., 2020), inspiring us that purine metabolic genes perform great potential as targets for HIGS to enhance the resistance of transgenic plants. Taken together, these results suggest that the AMP *de novo* pathway is important for growth and essential for pathogenicity.

In *C. albicans*, *GUA1* is conditionally essential and can be bypassed when exogenous adenine is enough and available (Jiang et al., 2010). In *C. neoformans*, loss of inosine monophosphate dehydrogenase (IMPDH) lead to the slow growth and virulence defects (Morrow et al., 2012). In plant pathogenic fungi, the *M. oryzae* Imd4 (MoImd4) is proved to partially recover the defect of *S. cerevisiae imd4* mutant, and its deletion mutant has a significant growth difference compared to the wild-type strain. Moreover, the invasive hyphae of *Moimd4* are defective in attenuated pathogenicity in *M. oryzae* (Yang et al., 2019). However, there is only one ortholog (FgImd1) of IMP dehydrogenase in *F. graminearum*, and the *Fgimd1* mutant does not affect the formation of infection cushion, but shows a differentiation block of invasive hyphae. Furthermore, exogenous GMP and guanine could fully recover the vegetative growth of *Fgimd1* mutant but do not rescue the defects of sexual reproduction, indicating that GMP *de novo* synthesis is distinct from AMP *de novo* pathway in the sexual reproduction stage (Sun et al., 2021). Intriguingly, IMPDH is found to be a DNA-binding transcriptional repressor in *Drosophila* by attenuating the expression of histone genes and E2f, a key driver of cell proliferation (Kozhevnikova et al., 2012). Considering that IMPDH binds a CT-rich ssDNA element in a sequence-specific manner in both *Drosophila* and *Escherichia coli* (Kozhevnikova et al., 2012), this ability is probably conserved from prokaryotes to eukaryotes and remains to be further investigated in fungi.

To date, Guk1 responsible for the conversion of GMP into GDP is only characterized in *M. oryzae*. There are two members of Guk1 in *M. oryzae*: MoGuk1 and MoGuk2. MoGuk1 is orthologous to the yeast Guk1 and essential for viability (Cai et al., 2017); MoGuk2 exhibited enzymatic activity, and its mutant is weakly reduced in vegetative growth, conidial germination, appressorial formation, but obviously reduced in sporulation, and pathogenicity (Cai et al., 2017). Moreover, *Moguk2* mutant could not produce perithecia (Cai et al., 2017). These results indicate that MoGuk2 is functional for GTP *de novo* synthesis and is important for plant infection in the rice blast fungus.

Finally, Ndk1 is recruited to synthesize end-products ATP and GTP (Amutha and Pain, 2003). Intriguingly, there is evidence showed that Ndk1 is involved in suppressing host oxidative bursts that triggered immunity in rice cells. The *Mondk1* mutant could form melanized appressoria and penetrate rice leaf sheath and onion epidermal cell surfaces successfully. However, invasive hyphae of *Mondk1* mutant are confined in rice cells of the initial inoculated site and could not extend to neighboring cells (Rocha and Wilson, 2020).

Together, disruption phenotypes of GMP *de novo* pathway are less severe than that of AMP *de novo* genes, suggesting that AMP *de novo* pathway plays more important roles in growth and virulence of plant pathogenic fungi. Moreover, ATP and GTP synthesis processes are rewarding to be lucubrated in virulence and suppression of plant immunity in the host.

## 5 Functions of purine salvage pathways in fungal pathogens

In *C. neoformans*, loss of the hypoxanthine-xanthine-guanine phosphoribosyltransferase Hpt1 in the salvage pathway had no phenotypes (Morrow et al., 2012). To date, functions of components catalyzing purine salvage pathway in plant pathogenic fungi are only characterized in *F. graminearum*. Unlike *S. cerevisiae*, *F. graminearum* has only a single gene (*FgHPT1*) orthologous to the *XPT1*. None of the deletion mutants of *Fghpt1*, *Fgacd3* (named *aah1* in *S. cerevisiae*), and *Fgacd14* (named *gud1* in *S. cerevisiae*) has detectable phenotypes (Sun et al., 2021), indicating that salvage synthesis of AMP/GMP is not important for growth, development and virulence. However, adding adenine could rescue the growth and sexual defects of the *Fgade12* mutant. Also, addition of exogenous guanine rescues the vegetative growth of the *Fgimd1* mutant (Sun et al., 2021), suggesting that purine salvage pathways are not defective in phenotypes but are indeed functional in *F. graminearum*.

Purine salvage pathway has dispensable role in growth, development and pathogenicity when the *de novo* pathway is functional in plant pathogenic fungi (Sun et al., 2021). Zrenner et al. (2006) suggested that due to its lower energy demand, the salvage pathway is likely used to maintain nucleotide pools in nongrowing cells, while *de novo* synthesis is predominantly utilized in proliferating cells (Zrenner et al., 2006). This may suggest that purine *de novo* synthesis is critical during stages of transition in pathogenic fungi, as new cells need to be rapidly proliferated, while the purine salvage pathway may supplement newly synthesized nucleotides to balance degradation processes in older cells.

Exogenous purines can be transported into intracellular space by purine transporters, which is a process required to balance nucleotide pools. Functional characterizations have been reported in several fungal organisms, for example, in *Aspergillus nidulans*, *S. cerevisiae*, and necrotrophic fungus *Botrytis cinerea* (Alguel et al., 2016; Daumann et al., 2016; Boswell-Casteel et al., 2018). Interestingly, the maize purine transporter LPE1 can complement the growth of *uapA uapC azgA* triple mutants on uric acid in *A. nidulans* (Argyrou et al., 2001), suggesting that the function of purine transporter is likely conserved among eukaryotes. In spite of these studies, purine transporters in plant pathogenic fungi are largely undefined, and further research is needed to unravel their roles in development and virulence.

## 6 Genes related to purine catabolic pathway in plant pathogenic fungi

Similar to its yeast orthologous gene *AMD1*, *ACD1* is dispensable for vegetative growth but significantly affects conidiation and sexual reproduction in *F. graminearum*. By heterologous expression, yeast *AMD1* partially recovers the defect of perithecia formation of *acd1* mutant (Sun et al., 2021). This result indicates that the AMP catabolic pathways may have the conservative functions during sexual reproduction in fungi. Notably, the *acd1* mutant is able to form abundant infection cushions similar to the wild-type strain during the stage of plant infection, but its invasive hyphae are blocked after penetration of host cells, suggesting that *ACD1* is not essential for the initial penetration and colonization but is important for the differentiation and growth of invasive hyphae in the wheat rachis (Sun et al., 2021). Additionally, exogenous IMP or GMP (but not AMP) can rescue the defects in perithecia formation and development for *acd1* mutant, but cannot recover the infectious defects. This significant difference in Acd1 during saprophytic and infectious growth suggests that purine catabolic metabolism maybe specifically regulated in plant infection. This is the first experimental evidence convincingly showing that purine metabolism has different roles during saprophytic and infectious growth in plant fungal pathogens. The stage-specific functions of the AMP deaminase gene maybe conserved in plant pathogenic fungi, and its regulation mechanism during the infectious stage needs to be disclosed and further elucidated.

In the hemibiotroph *Colletotrichum graminicola*, the maize anthracnose fungus, the purine degradation genes *ALA1* and *URE1* encoding ureases are required for appressorial penetration and full virulence. The urease inhibitor acetohydroxamic acid (AHA) significantly protects the host, and interferes the incasice process of hemibiotrophs and biotrophs fungi, and oomycetes (Benatto Perino et al., 2020). The inhibition of purine catabolic pathway could be a choice for fungicide exploration and application to control diseases caused by plant fungal pathogens and oomycetes. Other components responsible for enzymatic steps of the catabolic pathway remain to be characterized to elucidate their roles in growth and virulence in plant pathogenic fungi.

## 7 Conclusion and perspectives

Overall, purine metabolic pathways play different roles in growth, conidiation, development, and infection in plant pathogenic fungi. Intriguingly, the genes encoding enzymes catalyzing the catabolic pathways showed stage-specific regulation of sexual and infectious growth in plant fungal pathogens. Notably, a few genes in salvage and catabolic pathways are absent or have a low identity of sequence similarity in *M. oryzae* in comparing with *F. graminearum*, indicating that the corresponding reactions are slightly different in these pathways between them, therefore, we recommend that more purine metabolic enzymes should be characterized in plant pathogenic fungi. Further investigation of metabolic enzymes, transporters, transcription factors, and crosstalk among purine metabolic pathways can lead to a better understanding of the purine metabolic network involved in the regulation of different development and infection processes.

## Author contributions

MS: Writing – original draft, Writing – review & editing. PD: Writing – review & editing. ZC: Writing – review & editing. JD: Writing – review & editing.
